# LETM1-Mediated K^+^ and Na^+^ Homeostasis Regulates Mitochondrial Ca^2+^ Efflux

**DOI:** 10.3389/fphys.2017.00839

**Published:** 2017-11-17

**Authors:** Shane Austin, Mojtaba Tavakoli, Christina Pfeiffer, Julia Seifert, Andrea Mattarei, Diego De Stefani, Mario Zoratti, Karin Nowikovsky

**Affiliations:** ^1^Department of Internal Medicine I and Comprehensive Cancer Center, Medical University of Vienna, Vienna, Austria; ^2^Department of Chemical Sciences, Università di Padova, Padova, Italy; ^3^Department of Biomedical Sciences, Università di Padova, Padova, Italy; ^4^Institute of Neuroscience (CNR), Padova, Italy

**Keywords:** LETM1, mitochondrial cation/proton exchange, mitochondrial volume homeostasis, potassium, sodium, calcium

## Abstract

Ca^2+^ transport across the inner membrane of mitochondria (IMM) is of major importance for their functions in bioenergetics, cell death and signaling. It is therefore tightly regulated. It has been recently proposed that LETM1—an IMM protein with a crucial role in mitochondrial K^+^/H^+^ exchange and volume homeostasis—also acts as a Ca^2+^/H^+^ exchanger. Here we show for the first time that lowering LETM1 gene expression by shRNA hampers mitochondrial K^+^/H^+^ and Na^+^/H^+^ exchange. Decreased exchange activity resulted in matrix K^+^ accumulation in these mitochondria. Furthermore, LETM1 depletion selectively decreased Na^+^/Ca^2+^ exchange mediated by NCLX, as observed in the presence of ruthenium red, a blocker of the Mitochondrial Ca^2+^ Uniporter (MCU). These data confirm a key role of LETM1 in monovalent cation homeostasis, and suggest that the effects of its modulation on mitochondrial transmembrane Ca^2+^ fluxes may reflect those on Na^+^/H^+^ exchange activity.

## Highlights

- Monovalent cation homeostasis is dysregulated upon LETM1 depletion
- K^+^/H^+^ exchange activity is decreased in LETM1 knockdown cells
- LETM1 depletion results in K^+^ accumulation in the mitochondrial matrix
- LETM1 knockdown does not affect expression of major mitochondrial Ca^2+^ transport modulators
- LETM1-regulated mitochondrial Ca^2+^ fluxes are dependent on Na^+^

## Introduction

The chemiosmotic theory is the cornerstone of the current model of mitochondrial energy conservation and whole cell bioenergetics. An essential feature of the chemiosmotic theory relies on the existence of mitochondrial cation/proton exchangers (third postulate) (Mitchell, 1966, 2011). These exchangers are understood to maintain mitochondrial matrix cation concentrations far from electrochemical equilibrium, thus setting mitochondrial volume at steady-state (Garlid, 1988a). Historically, two monovalent cation/proton electroneutral exchange activities have been described: one highly selective for Na^+^ (Na^+^/H^+^ exchanger, NHE), the other more promiscuous (accepting K^+^, Na^+^, and Li^+^). Given the ion composition of the cytoplasm and mitochondrial matrix, this latter non-selective antiporter has been generally considered to function as a K^+^/H^+^ exchanger (KHE) (Nakashima and Garlid, 1982).

K^+^/H^+^ exchange is central to mitochondrial volume homeostasis, given that K^+^ is the most abundant intracellular monovalent cation. This is pointed out in some of the earliest accounts of the process (Garlid, 1988a). The properties of the electroneutral KHE, compensating K^+^ efflux by H^+^ intake, have been extensively studied in the 1980s using isolated mitochondria (Nakashima and Garlid, 1982; Brierley et al., 1984; Garlid et al., 1986). KHE activity is stimulated by decrease of medium osmolarity and increase of pH. Equally, inhibition by Mg^2+^, quinine and inhibitors of the respiratory chain is well described (Brierley et al., 1984; Garlid et al., 1986; Welihinda et al., 1993). Remarkably, K^+^ efflux rates depend not only on the K^+^ and H^+^ gradients, but also on the magnitude of the transmembrane potential. This has been ascribed to the modulation of the active conformation of the antiporter by ΔΨ, and it is not in contradiction with the key feature of electroneutrality (Bernardi and Azzone, 1983).

The mitochondrial NHE also has a role in ion homeostasis (Nath and Garlid, 1988), and it was initially characterized as involved in the regulation of mitochondrial Ca^2+^ (Garlid, 1988b). The NHE is insensitive to classical KHE inhibitors (Mg^2+^, quinine or DCCD) (Nakashima and Garlid, 1982; McCormack and Denton, 1986). As mentioned, a key functional difference between the two activities is that while the NHE is selective for Na^+^ and Li^+^ over K^+^ (Mitchell and Moyle, 1969), the KHE discriminates poorly between K^+^ and other monovalent cations (Nath and Garlid, 1988). A differential regulation of KHE-mediated electroneutral cation fluxes is however provided by Mg^2+^, which can block K^+^ but not Na^+^ antiport (Bernardi, 1999). In contrast, the NHE is competitively inhibited by Li^+^ but appears not to be regulated by any other cations (Nath and Garlid, 1988).

To date the molecular identity of the KHE remains unknown. Garlid and colleagues identified an 82 kDa protein able to mediate K^+^/H^+^ exchange in liposomes, and proposed it as the mitochondrial KHE (Li et al., 1990). A decade later, the protein Mdm38p was identified in yeast to be responsible for regulating mitochondrial volume (Nowikovsky et al., 2004). This protein possesses several properties expected of the mitochondrial KHE and its mammalian homolog, LETM1, has been shown to be necessary and sufficient to control K^+^/H^+^ exchange (Froschauer et al., 2005). Strong evidence of an essential role of LETM1 (Blomen et al., 2015; Wang et al., 2015) is in line with the notion of the vital function in maintaining the mitochondrial volume homeostasis, as postulated by P. Mitchell and with the finding that loss of LETM1 on one allele is sufficient to determine (or cause) the Wolf Hirschhorn syndrome (WHS). However, the identity of LETM1 as the KHE itself or a key factor of mitochondrial K^+^/H^+^ exchange has been challenged by reports that it operates as a mitochondrial Ca^2+^/H^+^ exchanger (Jiang et al., 2009, 2013; Tsai et al., 2014). Jiang and co-workers performed a genome-wide RNAi screen using *Drosophila* cells expressing the fluorescent mitochondrial-pericam Ca^2+^ probe (mt-pericam) (Jiang et al., 2009). In these assays, mt-pericam reported *in situ* Ca^2+^ levels in intact cells upon addition of thapsigargin, a SERCA blocker known to increase cytosolic Ca^2+^. These measurements as outlined in the Supplementary Methods (Jiang et al., 2009, SOM Text) were performed in Krebs-Ringer-HEPES buffer which contained 125 mM NaCl. The article demonstrated that cells with impaired LETM1 expression had reduced mt-pericam fluorescence.

Ca^2+^, which purportedly regulates mitochondrial kinases and dehydrogenases, is readily taken up in energized mitochondria by the MCU complex (Baughman et al., 2011; De Stefani et al., 2011). Yet, excess matrix Ca^2+^ would cause the calcification of the organelle, a condition that is not observed *in vivo*. As it is the case also for K^+^, efflux systems in the mitochondrial inner membrane must operate to prevent Ca^2+^ from distributing according to the transmembrane potential (Δψ). Mitochondrial Ca^2+^ release has been shown to be induced by Na^+^ and mediated by a Na^+^/Ca^2+^ antiporter, which exchanges mitochondrial Ca^2+^ for external Na^+^ (Carafoli et al., 1974). A NHE is then required to release matrix Na^+^ in exchange for external H^+^, allowing the Na^+^/Ca^2+^ exchange to continue (McCormack and Denton, 1986). While NCLX (SLC8B1) has been identified as the mitochondrial Na^+^/Ca^+^ antiporter (Palty et al., 2010), an additional Ca^2+^ efflux pathway is thought to be provided by a Ca^2+^/H^+^ exchanger (CHX) (McCormack and Denton, 1986; Kamer and Mootha, 2015). It has been proposed that this exchange is carried out by LETM1 (Jiang et al., 2009, 2013; Tsai et al., 2014). The interplay of NCLX and CHX in mitochondria Ca^2+^ dynamics is essential, as was recognized as early as the 1970s and continues to be well appreciated today (Carafoli, 1979; Kamer and Mootha, 2015). In fact, both components are fundamental players in any model of the mitochondrial Ca^2+^ cycle. This is so despite the molecular identity of NCLX having only recently been determined and the CHX function of LETM1 being still debated (Palty et al., 2010; Nowikovsky and Bernardi, 2014). Given this background, we hypothesized that LETM1/KHE may influence electroneutral Ca^2+^ transport indirectly via its regulation of monovalent cation homeostasis. Here we investigate the effect of LETM1 knockdown on monovalent cation exchange and mitochondrial Ca^2+^ transport activities and clarify LETM1 functions under physiological conditions.

## Methods

### Antibodies

Antibodies used were as follows: LETM1 (H00003954-M03, Abnova, Taipei, Taiwan), GAPDH (sc25778, Santa Cruz Biotechnology, Dallas, TX). Secondary HRP conjugated AffiniPure antibodies were from Jackson Immunoresearch (West Grove, PA): goat anti-mouse (115-035-008) and goat anti-rabbit (111-035-008).

### Reagents

All reagents were from Sigma Aldrich (Vienna, Austria) unless otherwise indicated. Thapsigargin was from Tocris (Bristol, UK) and puromycin from Invivogen (San Diego, CA). All buffers as formulated were completed with MilliQ (Billerica, MA) ddH_2_O.

### Primers and plasmids

Primers are listed in the Table S1. All primers were from Microsynth (Balgach, Switzerland). All shRNA constructs were obtained from Origene Technologies (Rockville, MD). Product numbers for control and constructs by gene are: LETM1 #1 TR311758A) and #2 (TR311758D). Sequences of shRNA constructs and targeted exons can be found in Table S2. pcDNA3 mtRFP was obtained as a generous gift from Prof. Tullio Pozzan (University of Padova, Italy).

### Cells

HeLa cells were a gift from E. Tomasich (Medical University of Vienna, Austria). All cells were maintained in DMEM (Gibco) supplemented with 10% (v/v) FBS (Gibco) and 1X penicillin/streptomycin/glutamine (Gibco). Cells were regularly screened for mycoplasma (MycoAlert Lonza kit).

### Stable cell generation

Cells were transfected with Turbofect (Invitrogen, Paisley, UK) according to manufacturer's instructions; 48 h post transfection the media was changed to growth media with puromycin (2 μg/mL). After a resistant population of cells was established, cells were maintained in growth medium containing puromycin (2 μg/mL).

### Immunoblotting

Cells were washed with PBS (Gibco) then harvested using lysis buffer (150 mM NaCl, 50 mM Tris (pH 8), 1% IGEPAL C360 and one tablet of protease inhibitors with EDTA (Roche) per 10 mL). Cell lysates were vortexed for 30 min at 4°C and then cleared by centrifugation at 15,000 g for 15 min, 4°C. Proteins were quantified by Bradford assay (Bio-Rad, Hercules, CA) using BSA as standard. Protein lysates were separated by SDS-PAGE and blotted onto 0.45 μm PVDF membranes (ThermoFisher Scientific, Rockford, IL). Membranes were immunoblotted as indicated and fat-free milk was used as blocking medium. Blots were quantified using the BioRad Image Lab (v5.2.1) software.

### qPCR

Total RNA was isolated from cells using TRIzol reagent (Invitrogen) according to the manufacturer's instructions. Total RNA was DNase I (Invitrogen) digested before reverse transcription using the High-Capacity cDNA Reverse Transcription Kit (AB Biosystems, Foster City, CA). Diluted cDNA was then used for quantitative PCR using the GoTaq qPCR master mix (Promega, Madison, WI) and the reaction was run on the StepOne Plus (AB Biosystems). Analysis was performed using the StepOne Plus software (v2.3) and Expression Suite (v1.0.3).

### Ca^2+^ uptake/efflux assays

Cell counting, harvesting and permeabilization were performed as in Wilfinger et al. (2016), with only minor modification. Permeabilized cells were studied in either intracellular like buffer I (ICL buffer I) (130 mM KCl, 10 mM Tris-MOPS (pH 7.4), 10 μM EGTA-Tris, 1 mM KPi, 5 mM malate, 5 mM sodium glutamate, 1 μM Ca^2+^ green 5N) or intracellular like buffer II (ICL buffer II) (130 mM KCl, 10 mM Tris-MOPS (pH 7.4), 10 μM EGTA-Tris, 1 mM KPi, 5 mM malate, 5 mM glutamic acid, 1 μM Ca^2+^ green 5N).Importantly, these buffers are identical, with the exception that ICL I contains sodium glutamate and ICL II glutamic acid. The pH of the ICL buffers was verified prior to measurements. Sequential additions of 10 μM Ca^2+^ (as CaCl_2_), 0.2 μM ruthenium red (RR) or 1.2 μM ruthenium 360 (R360), 10 mM Na^+^ (as sodium acetate, NaOAc) and 2 μM FCCP were as indicated in **Figure 4**. Ca^2+^ uptake/release measurements were performed at 25°C on a LS55 spectrofluorometer (Perkin Elmer) with the following parameters, λ_ex_ = 505 nm, λ_em_ = 535 nm, slit width: 2.5 nm.

### Mitochondrial swelling assays

Isolated mitochondria (Frezza et al., 2007) were deenergized by incubation with 5 μM antimycin A for 10 min at RT (25°C). Mitochondria were pelleted and resuspended in isotonic isolation buffer (10 mM Tris-MOPS (pH 7.4), 1 mM EGTA-Tris (pH 7.4), 200 mM sucrose) at a concentration of 1 mg/mL and placed in a cuvette. In the case of KOAc-induced swelling, mitochondria were additionally depleted of Mg^2+^ immediately prior to measurement by incubation with 1 μM A23187 and 1 mM EDTA to eliminate Mg^2+^, the blocker of the KHE. To measure KOAc- or NaOAc-induced passive swelling, KOAc (as described in Nowikovsky et al., 2004: 55 mM KOAc, 5 mM TES, 0.1 mM EDTA) or NaOAc (55 mM NaOAc, 5 mM TES, 0.1 mM EDTA) buffer, respectively, was added and the optical density changes were immediately recorded at 25°C using an UltroSpec 1100 Pro spectrophotometer (Biochrom, Cambridge, UK). Swelling rate was quantified by fitting the raw swelling data for one-component exponential decay as shown, and the *k* value was used to quantify the rate of the process.

### Mitochondrial membrane potential

Cells (2 × 10^8^) were harvested and permeabilized as described in Wilfinger et al. (2016). Rhodamine123 (Molecular Probes) was used as ΔΨ probe at a concentration of 0.15 μM in ICL buffer II. Mitochondria were energized with glutamate (5 mM) and malate (5 mM), maximal mitochondrial depolarization was achieved by the addition of FCCP (1 μM). Measurements were performed at 37°C on a LS55 spectrofluorometer with the following parameters: λ_ex_ = 503 nm, λ_em_ = 523 nm, slit width: Ex-3.5 nm, Em-2.5 nm.

### Mitochondrial potassium indicator

The potassium probe mitoPOP was used to monitor the mitochondrial K^+^ concentration. The probe was tested for its pH and K^+^ sensitivity as follows: pH sensitivity was measured by dissolving 10 μM mitoPOP in a solution containing 150 mM KCl and 25 mM MES (pH 6.5), HEPES (pH 7 and 7.4) or TRIS (pH 8 and 8.5). Fluorescence was recorded in a microplate reader (Perkin Elmer Envision) using 485/10 nm and 535/30 nm bandpass filters for excitation and emission, respectively. Raw fluorescence values were blank subtracted, normalized on neutral pH and plotted against pH values. In parallel, K^+^ sensitivity was tested by dissolving 10 μM mitoPOP in a solution containing 25 mM HEPES (pH 7) and 0, 150, or 300 mM KCl at 25°C. Raw fluorescence values (measured as described above) were blank subtracted, normalized on K^+^-free medium and plotted against [K^+^].

Cells (30,000/well) were loaded with mitoPOP (1 μM) for 45 min at 37°C under cell culture conditions in modified Krebs-Ringer buffer (135 mM NaCl, 5 mM KCl, 1mM MgSO_4_, 0.4 mM K_2_HPO_4_, 20 mM HEPES, 5.5 mM glucose, 1 mM CaCl_2_, pH = 7.4). Cells were washed once with Krebs-Ringer buffer and then incubated for 5 min in 140 mM Tris-KCl (pH 7.4). Thereafter increasing K^+^ or sucrose concentrations were added as indicated for the *in situ* response curve. Measurements were performed on a TriStar plate reader (λ_ex_:485 nm, λ_em_:535 nm) at 25°C. Cells were subsequently counted by Trypan blue exclusion to normalize fluorescence readings to cell number. As a double control, to directly normalize the results to the amount of mitochondria independently of potentially variable cell numbers, cells plated in duplicates were analogously loaded with MitoTracker green (50 nM, Molecular Probes) at 37°C for 30 min. The staining was washed once, the fluorescence intensity quantified as above in KCl buffer and used to normalize mitoPOP fluorescence values.

### Microscopy

Scrambled shRNA-transfected (control) HeLa cells were seeded onto an Ibidi μ-slide (Martinsried, Germany) and transfected as previously described with pcDNA3 mtRFP and stained with mitoPOP for the last 45 min prior to visualization. The cells were visualized and images acquired on an LSM780 confocal microscope. Microscope, objective and aperture specifications may be found in Wilfinger et al. (2016).

### Statistical testing

All statistical analyses were performed using GraphPad (La Jolla, CA) Prism v6 for Windows. Similarly, all graphs were generated with GraphPad Prism. One way ANOVA with Dunnett's posttest was performed in each case to assess significance. Individual *p*-values are indicated in the figure legends.

## Results

### LETM1 knockdown does not affect MCU and NCLX1 expression

**Figure 1:**
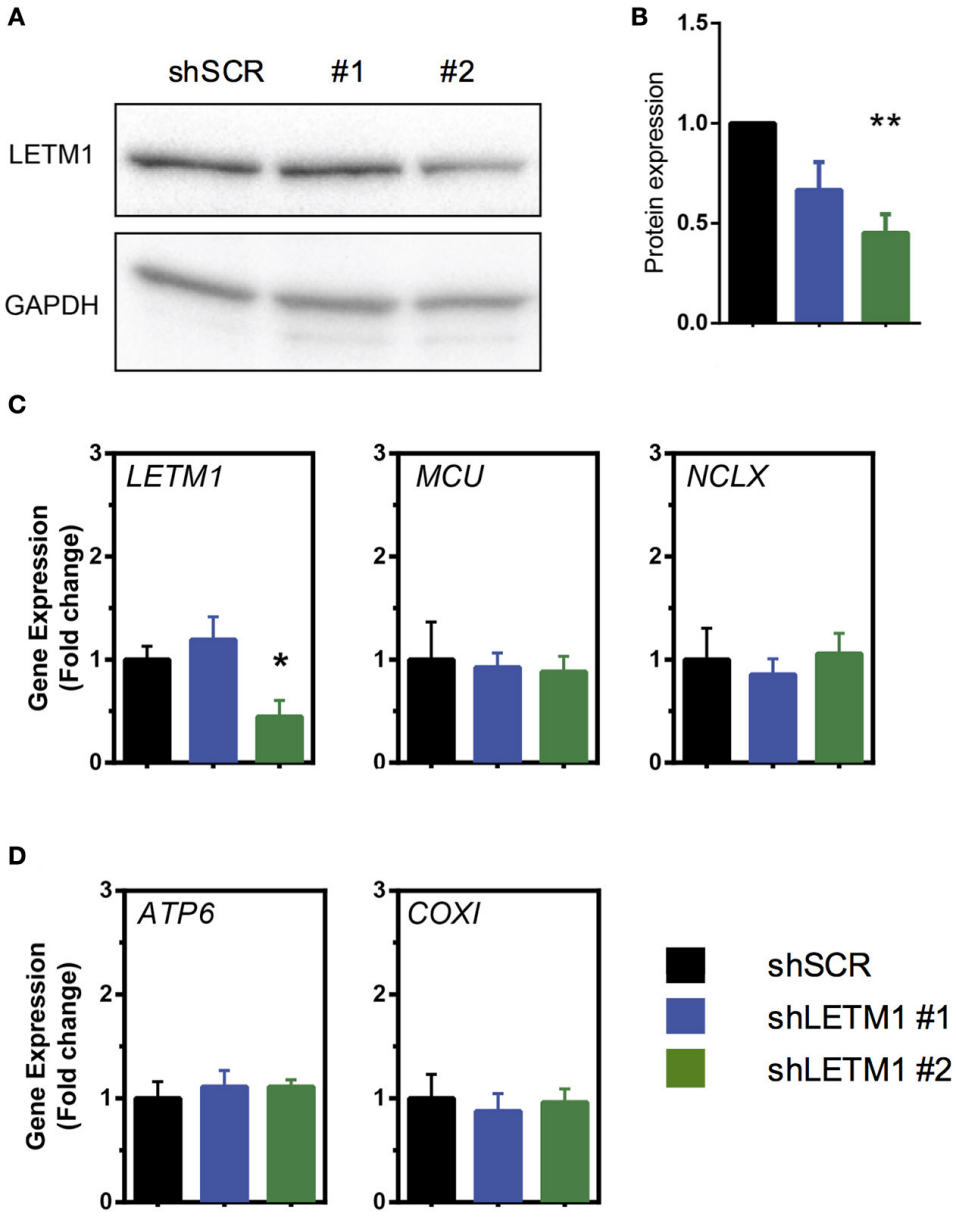
Mitochondrial Ca^2+^ modulators are unaffected by LETM1 expression. **(A)** Immunoblot of scrambled control (SCR) and LETM1 knockdown cells (#1, #2) for LETM1 and GAPDH as indicated. **(B)** Densiometric quantification of five independent immunoblots of samples as shown in **(A)**. Data shown are mean LETM1 expression relative to loading control ± SEM (*n* = 5). ^**^p < 0.01, one way ANOVA with Dunnett's multiple comparisons test. **(C)** Gene expression of (left to right as indicated): LETM1, MCU, NCLX (SLC8B1) in shSCR control and shLETM1 cells. All data are mean gene expression relative to *TBP* of *n* = 4–5 independent experiments ± SEM. ^*^p < 0.05, one way ANOVA with Dunnett's multiple comparisons test. **(D)** Gene expression of mitochondrially encoded genes (left to right as indicated): *ATP6, COXI*, compared to a nucleus-encoded control (*18S*) in shSCR control and shLETM1 cells. All data are mean gene expression relative to *TBP* of *n* = 4 independent experiments ± SEM.

To address the role of LETM1 in mitochondrial K^+^ and Ca^2+^ release, we stably transfected HeLa cells with shRNAs targeted against LETM1, or randomly scrambled constructs. HeLa cell lines with LETM1 knockdown of varying efficiency were obtained. We observed that the most efficient viable knockdown had a 50% reduction in gene expression when compared to scramble controls (shSCR). This was achieved with shRNA LETM1 #2, while for another construct, shRNA LETM1 #1, no significant reduction was observed (Figure 1C). However, surprisingly, at the protein level both knockdown cell lines showed decreased LETM1 expression, with shLETM1 #2 proving more efficient than shLETM1 #1 (Figures 1A,B). As mentioned before, WHS patients have only one copy of LETM1, hence a reduction of 50% is clinically relevant (Hart et al., 2014). We further asked if reduced LETM1 expression altered the expression of known mitochondrial Ca^2+^ transporters, the mitochondrial Ca^2+^ uniporter (MCU) or NCLX (SLC8B1), and found both genes had comparable transcription levels regardless of LETM1 expression (Figure 1C). To further exclude that LETM1 KD may lead to loss of mitochondrial DNA and reduced mitochondrial mass, we compared the expression of the mitochondrially-encoded genes *COX1* and *ATP6* to that of a nuclear gene (*18S*) in our shLETM1 and control cells, and found no differences (Figure 1D).

### Mitochondrial K^+^ and Na^+^ efflux is reduced upon LETM1 knockdown

**Figure 2:**
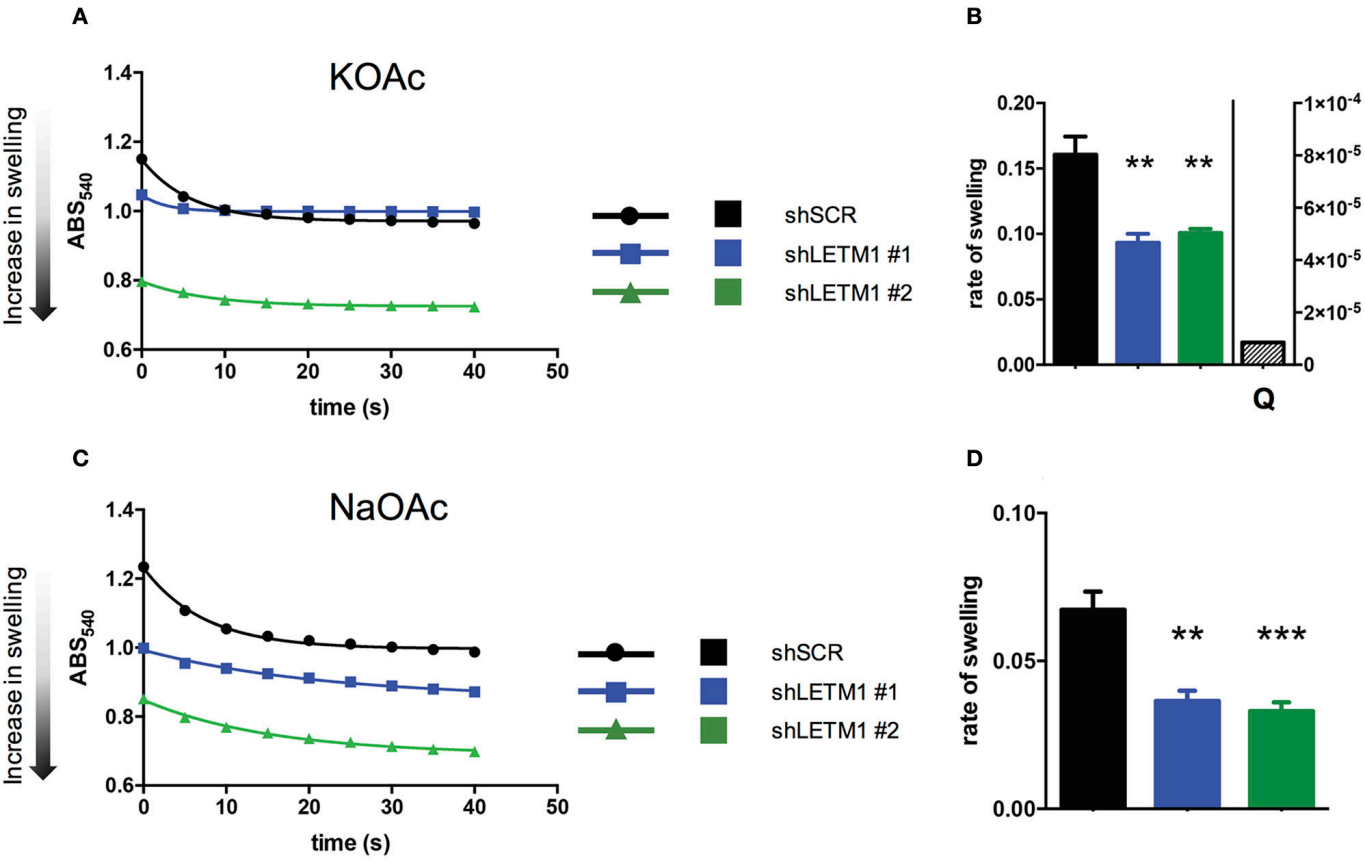
LETM1 expression regulates mitochondrial KHE. **(A)** Representative trace of KOAc induced swelling in shSCR (black circles) and shLETM1 (#1 blue squares, #2 green triangles) isolated mitochondria. The increase of matrix volume corresponds to the decrease of optical density, as indicated by the arrow. **(B)** Quantification of three independent swelling experiments as shown in **(A)**. Control demonstrating inhibition of swelling with quinine (shSCR + Q- quantified on right axis) is the average of two independent experiments with 1 mM quinine in swelling buffer. Data are means ± SEM (*n* = 3). ^**^p < 0.01, one way ANOVA with Dunnett's multiple comparisons test. **(C)** Representative trace of NaOAc induced swelling in shSCR (black circles) and shLETM1 (#1 blue squares, #2 green triangles) isolated mitochondria. **(D)** Quantification of four independent swelling experiments as shown in **(C)**. Data are means ± SEM (*n* = 4). ^**^p < 0.01, ^***^p < 0.005 one way ANOVA with Dunnett's multiple comparisons test.

So far, LETM1-dependent KHE activity has been assessed in *S. cerevisiae*, a yeast species that lacks mitochondrial Ca^2+^ transport systems. Using light scattering-based assays, yeast mitochondria devoid of LETM1 were shown to be refractory to KOAc-induced swelling, indicating reduced KHE activity and matrix K^+^ overload (Nowikovsky et al., 2004). To date, light scattering monitoring of passive swelling represents the most reliable way to evaluate KHE activity (Nakashima and Garlid, 1982; Garlid et al., 1986). Incubation of isolated non-respiring mitochondria in KOAc results in a rapid diffusion of acetic acid into the matrix where it deprotonates. The dissociation of H^+^ from AcO^−^ leads to the acidification of the matrix catalysing the uptake of K^+^ in exchange for H^+^. The matrix accumulation of K^+^ acetate induces matrix swelling which is seen as decreased of scattered light or optical density. Here, we applied the light scattering approach to isolated mitochondria from HeLa cells, and monitored for the first time the changes in optical density (OD) of suspensions of control and LETM1 KD mitochondria (Figure 2).

To reveal changes in the activity of the mitochondrial KHE we used hypotonic KOAc buffer, and deenergized mitochondria depleted of Mg^2+^, a known inhibitor of KHE activity. Incubation of isolated non-respiring mitochondria in KOAc leads to the acidification of the matrix, catalysing the uptake of K^+^ in exchange for H^+^. The matrix accumulation of K^+^ induces matrix swelling which is seen as decreased scattered light or optical density. In general, while a rapid and high amplitude swelling is expected from mitochondria with functional KHE, a reduction of KHE activity will be reflected by lesser swelling. We observed that shLETM1 #1 and #2 mitochondria had slower and decreased swelling as compared to shSCR controls, as reflected by a reduced and slower decrease of scattered light. The decrease of OD was inhibited by quinine, the inhibitor of KHE, confirming that the changes in OD were due to KHE activity (Figures 2A,B). Moreover, the matrix volume of shLETM1 mitochondria was increased as compared to that of shSCR mitochondria, as indicated by a lower OD from the beginning of recording, suggesting a higher content of osmolytes (Figure 2A). The mitochondrial Na^+^/H^+^ exchange activity was assayed in a similar swelling assay. In this case, Mg^2+^ depletion was not carried out and Na^+^ fluxes could not be inhibited by quinine (Bernardi, 1999). We obtained qualitatively identical results: both shLETM1 knockdown mitochondria displayed decreased swelling (Figures 2C,D). The data collected here using well-established and reliable methods, establish that LETM1 downregulation results in a significant decrease of proton exchange for each monovalent cation, K^+^ or Na^+^.

### K^+^ is accumulated in the matrix of LETM1-depleted mitochondria

**Figure 3:**
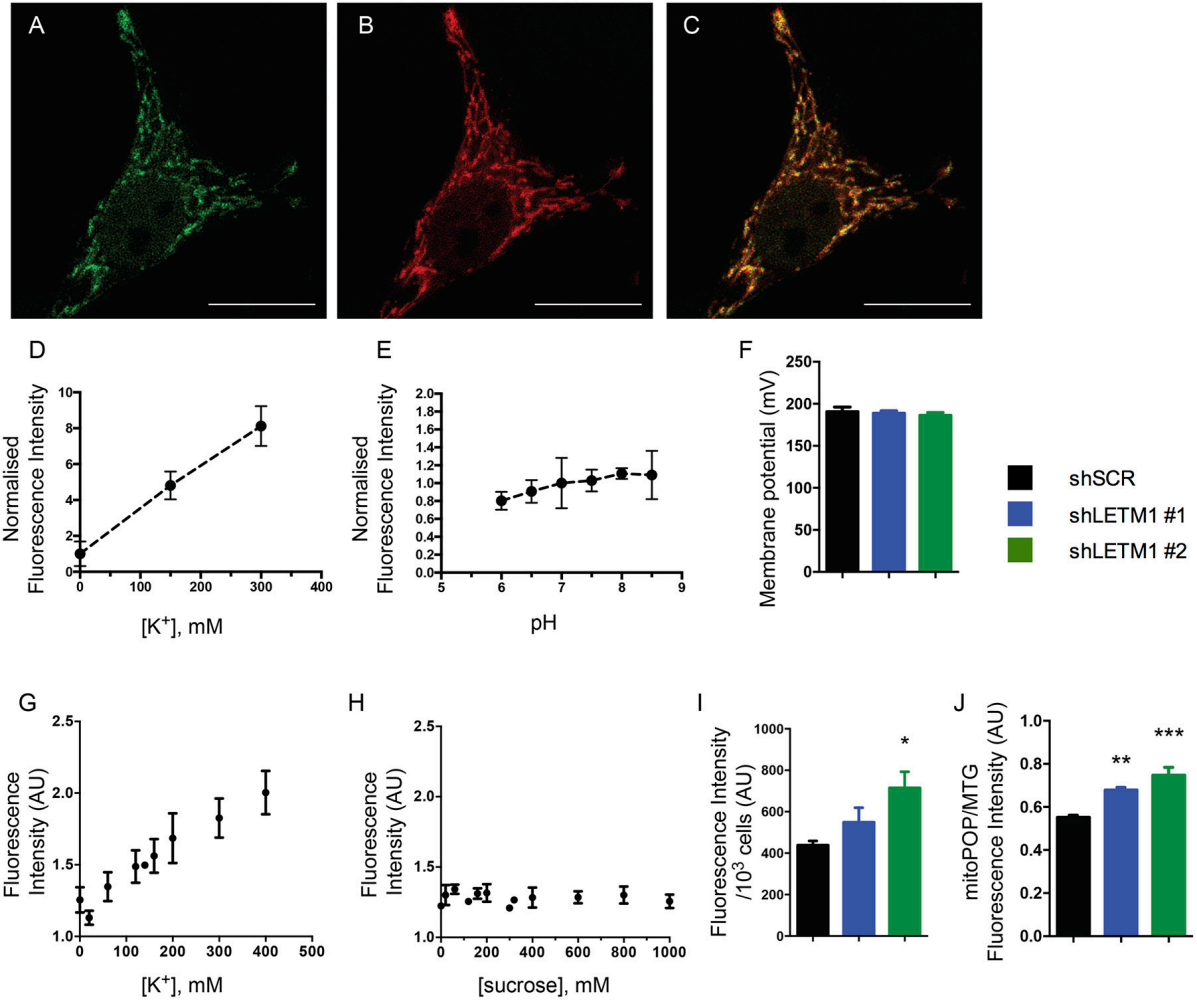
LETM1 depletion results in K^+^ accumulation in the matrix. **(A–C)** Intact HeLa control cells expressing mtRFP **(B)** were loaded with mitoPOP **(A)**, to demonstrate mitochondrial localization of the probe merged image **(C)**. Confocal microscopy images are representative of cells in 2 independent experiments. Scale bar: 20 μm in all images. **(D)** Fluorescence intensity (normalized on K^+^-free medium) of the mitoPOP probe in response to different [K^+^]. Data are means ± SD (*n* = 3). **(E)** Fluorescence intensity (normalized on neutral pH) of the mitoPOP probe at different pH values. Data are means ± SD (*n* = 3). **(F)** Mitochondrial membrane potential of permeabilized HeLa control (shSCR) and LETM1 knockdown (shLETM1 #1, #2) cells. Data are means ± SD (*n* = 3). **(G)** Response curve obtained by addition of KCl as indicated to individual wells containing intact HeLa control cells loaded with mitoPOP indicator. Data are means ± SEM (*n* = 3). **(H)** Response curve obtained by addition of sucrose as indicated to individual wells containing intact HeLa control cells loaded with mitoPOP indicator. Data are means ± average deviation (*n* = 2) conducted in technical duplicates. **(I)** Normalized mitoPOP fluorescence intensity of intact HeLa control (shSCR) and LETM1 knockdown (shLETM1 #1, #2) cells. Fluorescence values were normalized to the cell number after measurement. Data are means ± SEM (*n* = 5). ^*^p < 0.05, one way ANOVA with Dunnett's multiple comparisons test. **(J)** MitoPOP fluorescence intensity of intact HeLa control (shSCR) and LETM1 knockdown (shLETM1 #1, #2) cells, normalized to MitoTracker Green fluorescence. Data are means ± average deviation (*n* = 4). ^**^p < 0.01, ^***^p < 0.005, one way ANOVA with Dunnett's multiple comparisons test.

Reduced KHE activity should result in accumulation of K^+^ in the matrix of energized mitochondria. To directly assess whether K^+^ indeed accumulated in LETM1 KD mitochondria, we employed a mitochondria-targeted fluorescent K^+^ probe (mitoPOP) which accumulates in the mitochondrial matrix due to the latter's negative electrical potential (Mattarei et al., unpublished results). The specific localization of the probe was confirmed by co-localization with mtRFP in shSCR control cells (Figures 3A–C). A titration of mitoPOP fluorescence as a function of [K^+^] (Figure 3D) and pH (Figure 3E) demonstrated the sensitivity of the probe for K^+^ changes in the desired concentration range and its insensitivity to pH (Figures 3D,E). In order to assess K^+^ differences in LETM1 KD cells, we preliminarily confirmed that the membrane potential was unaffected by LETM1 KD (Figure 3F) as previously reported (Doonan et al., 2014). Significant changes of the *in situ* mitochondrial transmembrane potential upon increasing additions of KCl up to 400 mM were excluded in experiments employing TMRM (Figure S1A). To demonstrate a response to K^+^ *in situ*, we then added KCl to intact shSCR mitoPOP-loaded HeLa cells. This did generate a response curve (Figure 3G). In comparison, adding sucrose to intact shSCR cells at increasing concentration had no effect on the mitoPOP fluorescence (Figure 3H and Figure S1B), thus excluding increasing osmotic pressure and confirming increasing K^+^ concentration as the reason for the observed fluorescence changes. Lastly, to assess if matrix K^+^ accumulation occurs in LETM1 KD cells, KD and control cells were exposed to a solution of 140 mM KCl after mitoPOP loading (Figures 3I,J). A 1.6-fold increase in fluorescence was observed in shLETM1 #2 cells in comparison to shSCR control cells, while shLETM1 #1 cells showed a more modest 1.3-fold increase. Together these results confirm that reduced KHE activity leads to matrix accumulation of K^+^ in LETM1-depleted cells.

### LETM1-dependent mitochondrial Ca^2+^ uptake and release is affected by the presence of Na^+^

**Figure 4:**
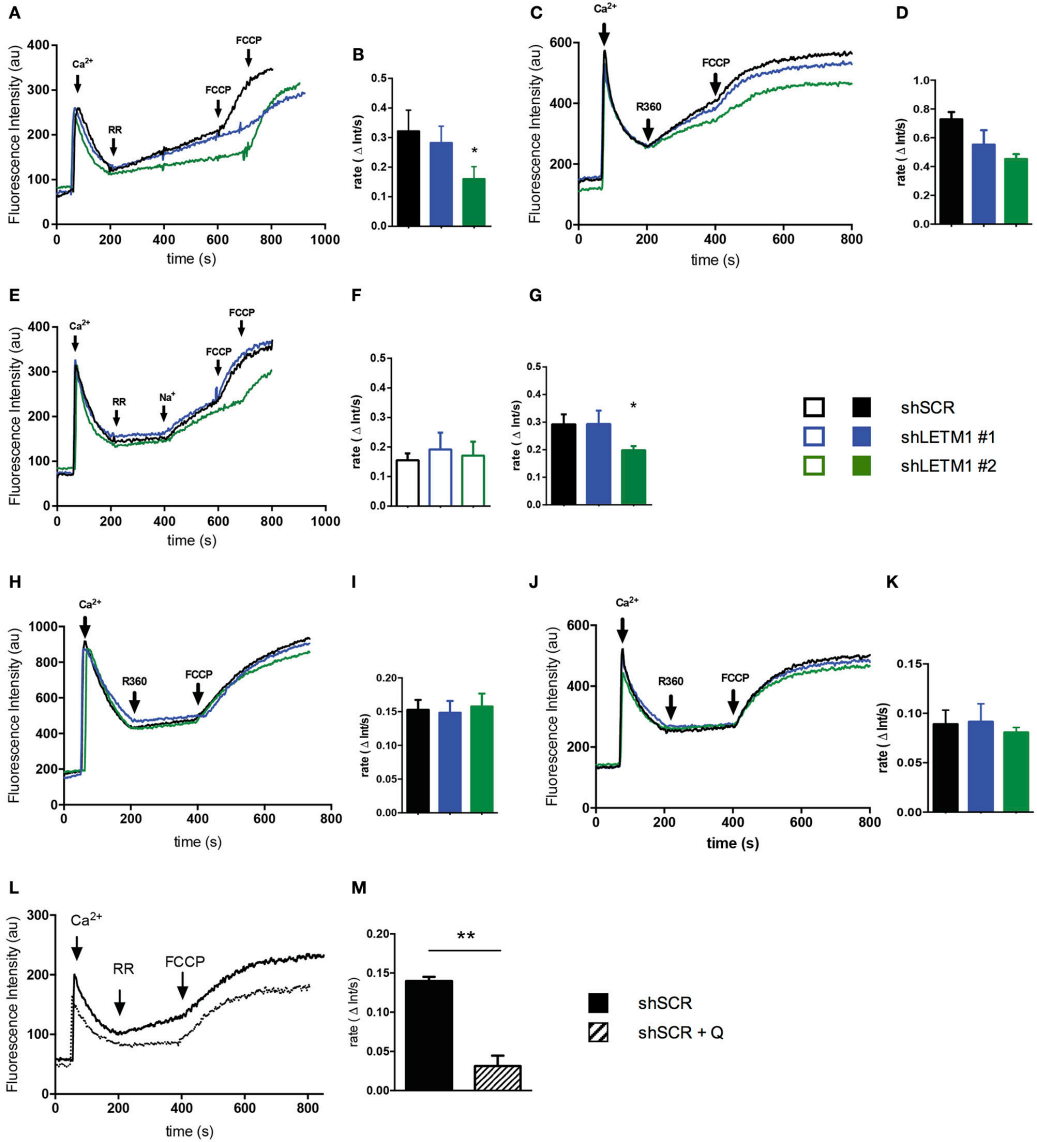
LETM1-mediated mitochondrial Ca^2+^ fluxes are Na^+^-dependent. **(A)** Representative trace of permeabilized scrambled control (shSCR) and LETM1 knockdown (shLETM1 #1, #2) cells in ICL buffer I (Na^+^ replete) containing Ca^2+^ Green 5N. Additions were made as indicated by arrows: Ca^2+^ (10 μM; 60 s), ruthenium red (RR) (0.2 μM; 200 s), FCCP (2 μM; 600/700 s). Recordings were performed at 25°C. **(B)** Quantification of Ca^2+^ efflux rate as shown in **(A)** between 200 and 400 s. Data shown are mean efflux rate ± SEM (*n* = 4). ^*^p < 0.05, one way ANOVA with Dunnett's multiple comparisons test. **(C)** Representative trace of permeabilized scrambled control (shSCR) and LETM1 knockdown (shLETM1 #1, #2) cells in ICL buffer I (Na^+^ replete) containing Ca^2+^ Green 5N. Additions were made as indicated by arrows: Ca^2+^ (10 μM; 60 s), ruthenium 360 (R360) (1.4 μM; 200 s) the active ingredient in ruthenium red, FCCP (2 μM; 400 s). Recordings were performed at 37°C. **(D)** Quantification of Ca^2+^ efflux rate as shown in **(C)** between 200 and 400 s. Data shown are mean efflux rate ± average deviation (*n* = 2). **(E)** Representative trace of permeabilized scrambled control (shSCR) and LETM1 knockdown (shLETM1 #1, #2) cells in ICL buffer II (Na^+^-free) containing Ca^2+^ Green 5N. Additions were made as indicated by arrows: Ca^2+^ (10 μM; 60 s), RR (0.2 μM; 200 s), Na^+^ (10 mM; 400 s), FCCP (2 μM; 600/700 s). Recordings were performed at 25°C. **(F)** Quantification of Ca^2+^ efflux rate as shown in **(E)** between 200 and 400 s. Data shown are mean efflux rate ± SEM (*n* = 5). **(G)** Quantification of Ca^2+^ efflux rate as shown in **(E)** between 400 and 600 s. Data shown are mean efflux rate ± SEM (*n* = 5). ^*^p < 0.05, one way ANOVA with Dunnett's multiple comparisons test. **(H)** Representative trace of permeabilized scramble control (shSCR) and LETM1 knockdown (shRNA #1, #2) as in **(A)** but in presence of CGP37175 an inhibitor of the Na^+^/Ca^2+^ exchanger (10 μM). **(I)** Quantification of Ca^2+^ efflux rate as shown in **(H)** between 200 and 400 s. Data shown are mean efflux rate ± SEM (*n* = 3–6). **(J)** Representative trace of permeabilized scramble control (shSCR) and LETM1 knockdown (shRNA #1, #2) as in **(C)** but in presence of CGP37175 as above. **(K)** Quantification of Ca^2+^ efflux rate as shown in **(J)** between 200 and 400 s. Data shown are mean efflux rate ± average deviation (*n* = 2). **(L)** Representative trace of permeabilized scrambled control (shSCR) cells treated with quinine (1 mM, dotted line) or vehicle control (ethanol, solid line) in ICL buffer I (Na^+^ replete) containing Ca^2+^ Green 5N. Additions were made as indicated by arrows: Ca^2+^ (10 μM; 60 s), RR (0.2 μM; 200 s), FCCP (2 μM; 400 s). Recordings were performed at 25°C. **(M)** Quantification of Ca^2+^ efflux rate as shown in **(B)**. Data shown are mean efflux rate ± SEM (*n* = 3). ^**^p < 0.01, unpaired students *t*-test.

Using our shLETM1 cells we performed mitochondrial Ca^2+^ uptake and release experiments similar in setup to those of Doonan et al. (2014) (Figure 4A). One pulse of Ca^2+^ was added to permeabilized LETM1 KD or control cells suspended in ICL buffer I containing the Ca^2+^ indicator Ca^2+^ 5N Green (Figure 4A, first arrow). Next, RR, an inhibitor of MCU, was added to block Ca^2+^ uptake and induce H^+^- and Na^+^-dependent Ca^2+^ release. As expected, a slow Ca^2+^ extrusion was observed (Figure 4A, second arrow). This was followed by a single pulse of the uncoupler FCCP to release sequestered mitochondrial Ca^2+^ (Doonan et al., 2014) (Figure 4A, third/fourth arrow). Consistent with the findings of Doonan et al., shLETM1 #2 cells had reduced mitochondrial Ca^2+^ efflux (Figures 4A–D), in a fashion that was dependent on LETM1 expression. The assays conducted at 25°C or 37°C showed comparable results. Based on the hypothesis that LETM1 embodies the KHE, which also transports Na^+^ (Nowikovsky et al., 2004; Froschauer et al., 2005), we next assessed Ca^2+^ transport in Na^+^-free buffer (ICL buffer II) (Figure 4E). In the absence of Na^+^, no change in Ca^2+^ efflux between cells expressing LETM1 at wild-type or knockdown levels was observed (Figures 4E,F). Next, the Na^+^-induced Ca^2+^ release was initiated by the addition of a Na^+^ pulse (Figure 4E, third arrow). As illustrated in Figure 4G, shLETM1 #2 showed reduced Na^+^-induced Ca^2+^ release. A comparison of the results obtained in the presence or absence of Na^+^ suggests that the effect of LETM1 on Ca^2+^ efflux depends on Na^+^ (Figures 4B,D,G vs. Figure 4F). For further validation, we subjected LETM1 control and knockdown cells to Ca^2+^ uptake/release assays in presence of the NCLX inhibitor CGP37157 using the Na^+^ replete buffer. This allows monitoring mitochondrial Ca^2+^ release that is not due to NCLX. As shown in Figures 4H–K. we found similar Na^+^-independent Ca^2+^ mitochondrial release activities in presence or absence of LETM1. The results were consistent at 25°C (4H, I) and 37°C (4J, K). To additionally evaluate the role of the KHE in mitochondrial Ca^2+^ fluxes using a pharmacological approach, we resuspended shSCR cells in Na^+^-replete buffer, and quantified Na^+^-dependent Ca^2+^ fluxes in control cells in the absence or presence of the KHE inhibitor quinine. As shown in Figures 4L,M, inhibition of the KHE severely decreased the Na^+^-dependent Ca^2+^ efflux in comparison to vehicle controls. These data support the hypothesis that the deregulation of mitochondrial monovalent cation homeostasis induced by KHE dysfunction reduces Ca^2+^ efflux.

## Discussion

The function of LETM1 in mitochondrial cation exchange has recently been a matter of debate. Studies in yeast, mammalian cell culture, *Trypanosoma, C. elegans* and *Drosophila* have demonstrated a role for this protein in K^+^/H^+^ exchange. While LETM1 has also been proposed to directly mediate mitochondrial Ca^2+^ uptake and efflux in *Drosophila*, mammalian cell culture and cell-free systems (Jiang et al., 2009, 2013; Doonan et al., 2014; Tsai et al., 2014), our data and those of others question this notion (Hashimi et al., 2013; De Marchi et al., 2014).

In this study, we show that decreased LETM1 expression substantially reduced not only K^+^/H^+^ exchange, which was expected, but also Na^+^/H^+^ exchange (Figures 2C,D), which is novel. These data support the earlier demonstration that LETM1 is an essential element of the mitochondrial KHE (Nowikovsky et al., 2004; Froschauer et al., 2005; Hashimi et al., 2013) and extend the range of functions of LETM1 to regulation of Na^+^-dependent processes. Both K^+^ and Na^+^ are fundamental for mitochondrial osmotic balance. The maintenance of their homeostasis is assured by mitochondrial exchange activities: KHE, which handles both K^+^ and Na^+^, and NHE, which is specific for Na^+^. Taking the demonstrated function of LETM1 in KHE into account, its discovery as a CHX in the *Drosophila* S2 screen was surprising (Jiang et al., 2009). We have already commented on the direction of a putative LETM1-mediated Ca^2+^/H^+^ exchange given a stoichiometry of Ca^2+^/2H^+^ (Nowikovsky and Bernardi, 2014). Thus, its function as a Ca^2+^ efflux mechanism must be considered. In direct contrast to the findings of the *Drosophila* screen and others (Jiang et al., 2013; Tsai et al., 2014), De Marchi et al. found that exogenous LETM1 expression led to a direct increase in K^+^-induced proton extrusion, a feature consistent with the KHE (De Marchi et al., 2014). This finding is coherent with a reduced K^+^/H^+^ exchange when LETM1 expression is decreased (this paper). A consequence of impaired KHE is K^+^ overload as has been demonstrated here and in yeast (Nowikovsky et al., 2004). Furthermore, recent data in *Trypanosoma* provide a substantial basis for the notion of an evolutionarily conserved function of LETM1 in KHE (Hashimi et al., 2013).

We have shown that LETM1 depletion reduces mitochondrial K^+^ and Na^+^ exchange for protons. Further, we have demonstrated that LETM1-depleted mitochondria have a reduced Ca^2+^ efflux in buffer containing Na^+^, while the efflux is unchanged in a Na^+^-free buffer. Of note, the Na^+^-independent Ca^2+^ release only accounts for a minor part of the mitochondrial Ca^2+^ efflux. Although the data of Doonan et al. are both convincing and technically sound, little consideration is given to the key observation that there is no difference in Ca^2+^/H^+^ fluxes after the addition of CGP37157, an inhibitor of the mitochondrial NCLX. Careful consideration of these data illustrates that the proposed LETM1-mediated Ca^2+^/H^+^ fluxes are unchanged when LETM1 expression is reduced (Doonan et al., 2014), in contrast to mitochondrial Ca^2+^/Na^+^ exchange. Contextually, this suggests LETM1 is not responsible for Ca^2+^/H^+^ fluxes *in vivo*. Furthermore, it has been reported that LETM1 does not mediate the bulk of electroneutral Ca^2+^ efflux in HeLa cells for which NCLX would instead be responsible (De Marchi et al., 2014). Thus, two possible explanations are apparent. One is that LETM1 may be directly involved in the regulation of the NCLX. However, our results indicate that LETM1 does not affect expression of NCLX, weakening this hypothesis. Alternatively, LETM1 may alter the efficiency of NCLX function by an indirect mechanism. Following the principles of non-equilibrium thermodynamics (De Stefani et al., 2016), high matrix Na^+^ concentration is expected to lower the activity of NCLX by decreasing the inward-directed Na^+^ chemical gradient. Furthermore, reduced monovalent cation translocation from the matrix would imply increased matrix alkalization, which might affect NCLX activity.

**Figure 5:**
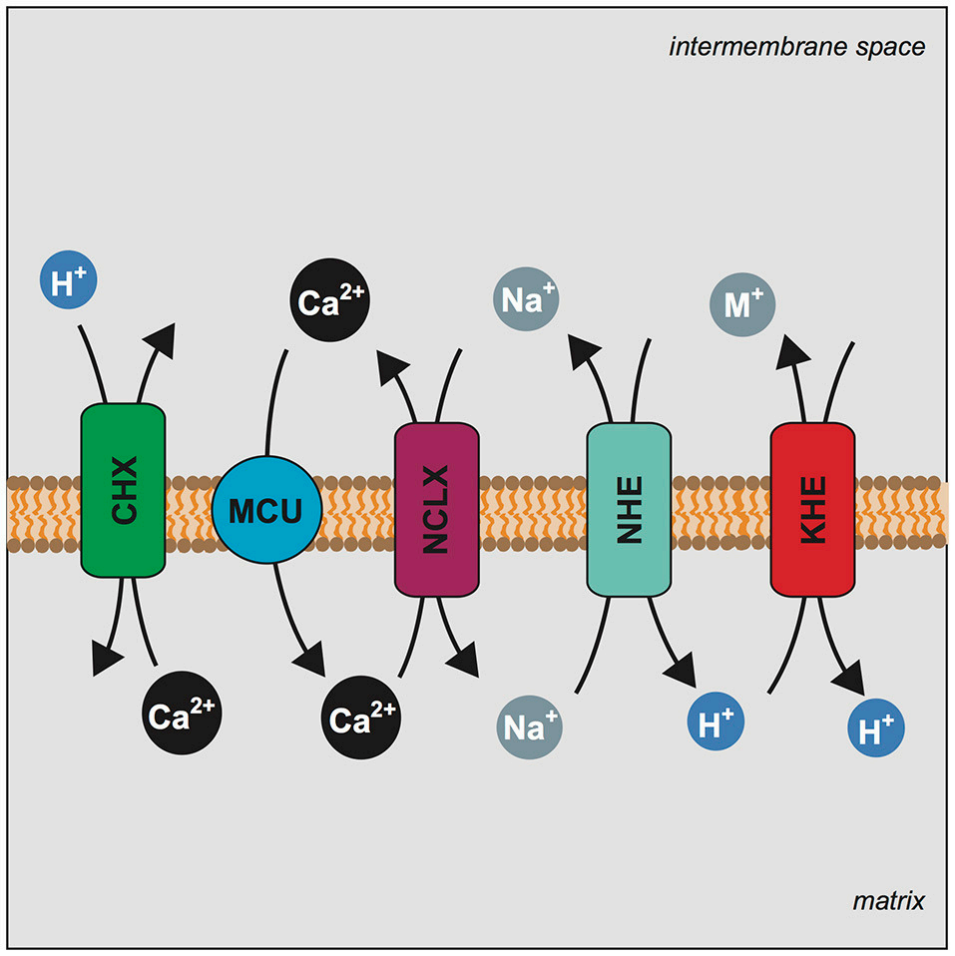
LETM1 dependent Ca^2+^ efflux is modulated by mitochondrial Na^+^ homeostasis. Mechanism illustrating the central players of the mitochondrial Ca^2+^ cycle and the proposed role of LETM1 in Ca^2+^ homeostasis. Stoichiometry of exchange is ignored for simplicity. Mitochondrial Calcium Uniporter (MCU) (blue circle) conducts the electrophoretic uptake of Ca^2+^ into mitochondria; this is regulated by members of the MCU core complex (EMRE, MICU1, MICU2). Mitochondrial Ca^2+^ efflux is executed by NCLX (SLC8B1) (purple rectangle) and the proposed Ca^2+^/H^+^ exchanger (CHX) (green rectangle). Na^+^ homeostasis is further regulated by a mitochondrial Na^+^/H^+^ exchanger (NHE) (teal rectangle), which would allow for the efflux of Na^+^. Additionally, mitochondrial K^+^/H^+^ exchange is executed by the KHE (LETM1) (red rectangle) a non-specific exchanger of monovalent cations (M^+^); the KHE can also extrude Na^+^. We propose that alteration of KHE activity by modulation of LETM1 levels affects Na^+^ homeostasis and this ultimately has an effect on Ca^2+^ release.

Based on the results presented above, we propose that LETM1 participates in Na^+^ cycling and thereby indirectly modulates Ca^2+^ fluxes. Indeed, Na^+^-driven Ca^2+^ release was proposed to be regulated by the combined activity of the NHE with the NCLX to dissipate excess Na^+^ or with the HCHX to dissipate excess H^+^ (Carafoli, 1979). Impairment of the KHE results in K^+^ and Na^+^ overload, the latter most likely slowing down the coordinated activities of NHE and NCLX, and thus resulting in decreased Ca^2+^ efflux. Less KHE activity most likely increases matrix alkalization, this also impinges on the NHE/NCLX. These simplified models, however, cannot account for the myriad of conditions, including changes of the matrix volume, increase of pH and the formation of Ca^2+^-phosphate precipitation, that play an important role in controlling the mitochondrial Ca^2+^ buffering capacity *in vivo*. Experimental systems using isolated cardiac mitochondria supported this notion (Blomeyer et al., 2013, 2016). Accordingly, changes in matrix Ca^2+^ buffering influence Ca^2+^ uptake and release, and thus Ca^2+^ sequestration, a non-negligible component of the mitochondrial Ca^2+^ cycle. Presumably, perturbations of matrix Na^+^ or pH also affect mitochondrial Ca^2+^ dynamics as highlighted earlier. Our results and model are in line with the idea of a mitochondrial Ca^2+^ cycle, which to maintain steady state, requires several components: MCU, NCLX, NHE, CHX, and KHE (Carafoli, 1979; McCormack and Denton, 1986; Kamer and Mootha, 2015) (Figure 5). Supporting this notion, addition of quinine mimics LETM1 depletion and thus shifts the balance of mitochondrial monovalent cations- increasing matrix K^+^ and likely matrix Na^+^. These perturbations significantly reduced mitochondrial Ca^2+^ efflux, reinforcing the importance of KHE activity for Ca^2+^ homeostasis. Despite our proposed mechanism of how LETM1 interplays with mitochondrial Ca^2+^ uptake and release via its role in mitochondrial monovalent cation homeostasis, the role of LETM1 EF hands in relation to osmotic regulation still needs to be addressed. Indeed, the presence of the EF hands has been reported to significantly affect Ca^2+^ uptake in mammalian systems (Doonan et al., 2014). While it is surprising that a mutation of the LETM1 EF hands can inhibit the Ca^2+^ uptake activity of MCU, one could speculate that this simple deletion elicits an unforeseen change in structure which prevents the activity or interaction with other crucial protein partners. However, these explanations are speculative and require further detailed investigation. Cell-free assay systems are indeed powerful tools to assess ion fluxes and protein activity. Nevertheless, these are simplified systems capable of reporting the activity of a single protein in isolation. Apparently, LETM1 can exhibit Ca^2+^/H^+^ exchange activity *in vitro*, as demonstrated by Tsai et al. or just recently by Shao et al. (Shao et al., 2016). Other factors crucial to osmotic balance such as the effects of other solute carriers and channels cannot be suitably assessed in these systems. Moreover, experiments to convincingly refute the role of LETM1 in regulating K^+^/H^+^ activities under physiologically relevant conditions are lacking. Whereas, experiments such as those by Hashimi et al. (2013) and De Marchi et al. (2014) continue to support the role of LETM1 in the KHE or question the involvement of LETM1 in Ca^2+^/H^+^ or Ca^2+/^Na^+^ exchange.

## Conclusions

We believe our results may well conclude the debate on the fundamental function of LETM1. We have shown that Na^+^-mediated mitochondrial Ca^2+^ efflux is susceptible to changes of LETM1-dependent KHE and NHE activity. This confirms once again the central function of LETM1 in mitochondrial osmotic balance and offers explanations for the changes in Ca^2+^ dynamics observed *in vitro*. Indeed, by modifying the activity of the KHE and monovalent cation homeostasis, alterations of LETM1 function will affect mitochondrial Ca^2+^ cycling independently of the expression level of any Ca^2+^ cycle component.

## Author contributions

SA and KN conceptualized and designed experiments. AM, DD, and MZ synthesized, validated and provided the mitoPOP probe. SA, MT, CP, and JS performed experiments, developed methods and analyzed data. SA, CP, and MT generated figures and performed statistical analyses. SA and KN wrote the manuscript. All authors approved the final version of the manuscript. KN conceived the project and supervised the study.

### Conflict of interest statement

The authors declare that the research was conducted in the absence of any commercial or financial relationships that could be construed as a potential conflict of interest.

## Supplementary material

Table S1 **Primers used in the study listed by gene which is targeted**.

Table S2 **List of shRNA constructs used, TR311758A is listed as shRNA #1 in this study, while TR311758D is listed as shRNA #2. The exon given is based on the sequence targeted in the accession number given**.

Figure S1 **Potassium (K^+^) and ΔΨ_m_ dynamics in HeLa cells. After loading with either mitoPOP (green traces) or TMRM (red traces), normalized fluorescence (F/F_0_) was recorded. When indicated, KCl **(A)** or sucrose **(B)** were added. Data are presented as mean ± S.D., *n* = 10 cells**.

## Abbreviations

ΔΨ: mitochondrial membrane potential
CHX: Ca^2+^/H^+^ exchanger
CsA: cyclosporin A
Da: Dalton
DCCD: dicyclohexylcarbodiimide
FCCP: Carbonyl cyanide 4-(trifluoromethoxy)phenylhydrazone
KD: knockdown
KHE: K^+^/H^+^ exchanger
KOAc: potassium acetate
MCU: mitochondrial Ca^2+^ uniporter
mPTP: mitochondrial permeability transition pore
NaOAc: sodium acetate
NHE: Na^+^/H^+^ exchanger
OD: optical density
SERCA: sarco/endoplasmic reticulum Ca^2+^-ATPase
RR: ruthenium red
R360: ruthenium 360
RT: room temperature
SD: standard deviation
SEM: standard error of the mean
TBP: TATA-box-binding protein
WHS: Wolf Hirschhorn syndrome.
