# Supplementary material for: LETM1-Mediated K+ and Na+ Homeostasis Regulates Mitochondrial Ca2+ Efflux
Source: Front Physiol. 2017 Nov 17;8:839. doi: 10.3389/fphys.2017.00839 (PMC5698270; doi:10.3389/fphys.2017.00839)
Supplement: Table S1 — Primers used in the study listed by gene which is targeted. [file Table1.PDF]

Supplementary Table Primers

	Primers	
**Gene**	**Fwd (5´-3´)**	**Rev (5´-3´)**
LETM1	GAGATTGTGGCAAAGGAAGC	CTTGGTGAGTGACTTCTTCTG
GHITM	GGCCTCAGTATGTCAAGGATAGA	GCGTTCTGCTGATTGCTATGG
MCU	TTCCTGGCAGAATTTGGGAG	AGAGATAGGCTTGAGTGTGAAC
NCLX (SLC8B1)	GGCATGGGCTCTGGGTTAC	TAACTGGCATGGGGCAGAAC
ATP6	CGCCACCCTAGCAATATCAA	TTAAGGCGACAGCGATTTCT
COXI	CGATGCATACACCACATGAA	AGCGAAGGCTTCTCAAATCA
TBP	TCTTTGCAGTGACCCAGCATC	GCAAACCAGAAACCCTTGCG
18S	CGTCACCCGTGGTCACCAT	CATTCGAACGTCTGCCCTATC
