# Supplementary material for: LETM1-Mediated K+ and Na+ Homeostasis Regulates Mitochondrial Ca2+ Efflux
Source: Front Physiol. 2017 Nov 17;8:839. doi: 10.3389/fphys.2017.00839 (PMC5698270; doi:10.3389/fphys.2017.00839)
Supplement: Table S2 — List of shRNA constructs used, TR311758A is listed as shRNA #1 in this study, while TR311758D is listed as shRNA #2. The exon given is based on the sequence targeted in the accession number given. [file Table2.PDF]

Supplementary Table Constructs

Gene	Accession number	Catalogue number	Sequence	Exon
LETM1	NM_012318.2	TR311758A	ACCAAGGAGAAGGAGGAGCTGGAGCTGCT	11
	NM_012318.2	TR311758D	AGGTGGCTGAGATTGTAGCAACACTGGAA	14
