# Supplementary figures and images for: LETM1-Mediated K+ and Na+ Homeostasis Regulates Mitochondrial Ca2+ Efflux
Source: Front Physiol. 2017 Nov 17;8:839. doi: 10.3389/fphys.2017.00839 (PMC5698270; doi:10.3389/fphys.2017.00839)

A

KCl

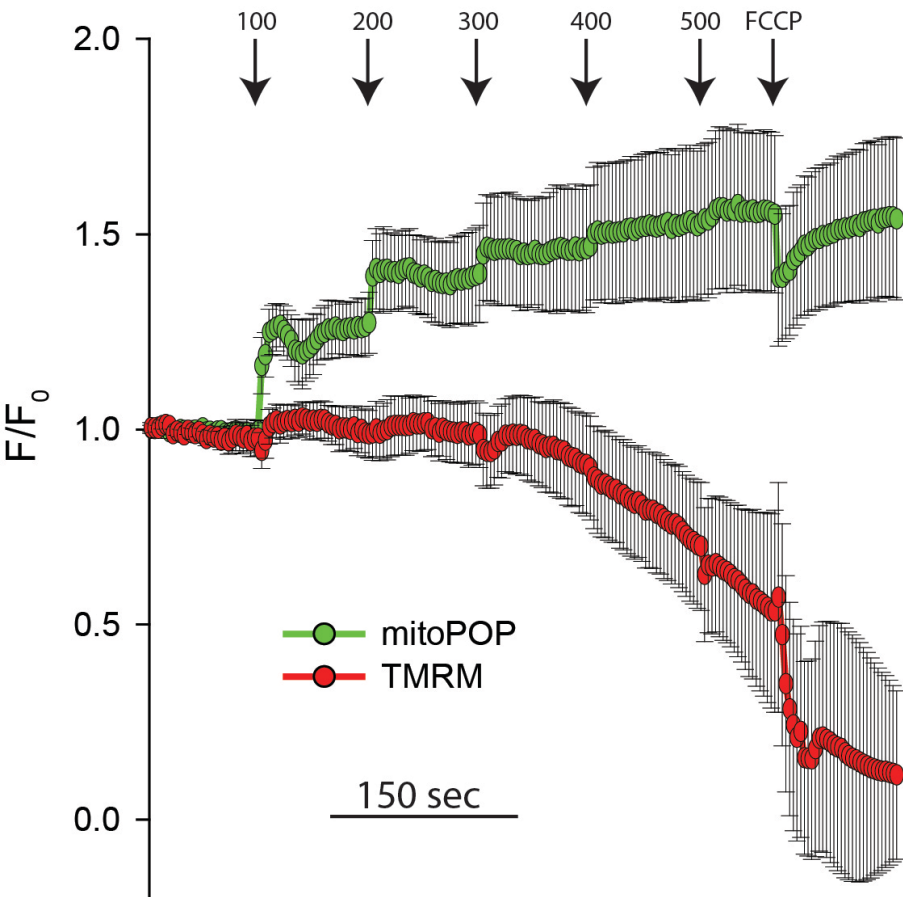

B

Sucrose

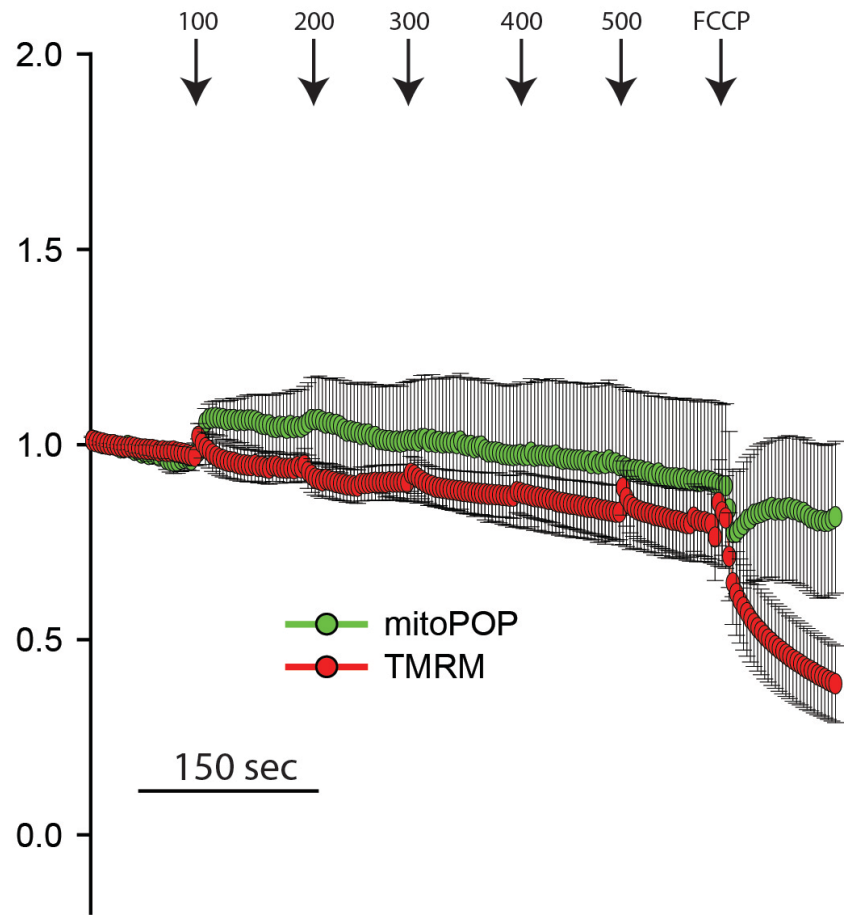

Supplement: Figure S1 — Potassium (K+) and ΔΨm dynamics in HeLa cells. After loading with either mitoPOP (green traces) or TMRM (red traces), normalized fluorescence (F/F0) was recorded. When indicated, KCl (A) or sucrose (B) were added. Data are presented as mean ± S.D., n = 10 cells. [file Image1.PDF]
