# Supplementary material for: LETM1-Mediated K+ and Na+ Homeostasis Regulates Mitochondrial Ca2+ Efflux
Source: Front Physiol. 2017 Nov 17;8:839. doi: 10.3389/fphys.2017.00839 (PMC5698270; doi:10.3389/fphys.2017.00839)
Supplement: Supplementary file 4 [file DataSheet1.docx]

Supplemental methods:

For mitochondrial K^+^ dynamics, HeLa cells were loaded with 1 μM mitoPOP for 1 hour at 37°C. For ΔΨ_m_ measurements, cells were loaded with 20 nM TMRM for 30 min at 37°C. Cells were then washed and placed in imaging buffer (250 mM sucrose, 1 mM EGTA, 25 mM Tris-Cl pH 7.4) and transferred to the imaging system. Every 30 frames, the indicated compounds were added. Images were acquired on a Zeiss Axiovert 200 microscope equipped with a 40x/1.3 N.A. PlanApochromat objective. Excitation was performed with a Deltaram V high speed monochromator (Photon Technology International) equipped with a 75W Xenon Arc lamp. Images were captured with a high sensitivity Evolve 512 Delta EMCCD (Photometrics). The system is controlled by Metamorph 7.5 and was assembled by Crisel Instruments. mitoPOP excitation was performed at 490 nm and emission was collected through a 510-560 bandpass filter. TMRM excitation was performed at 560 nm and emission was collected through a 590–650 nm bandpass filter. Images were acquired every 3 seconds with 300 or 100 milliseconds exposure times for mitoPOP and TMRM respectively. Images were background corrected and processed using the Fiji distribution of ImageJ.
